# Salvage VRAM Flap Reconstruction After Singapore Flap Failure Following Abdominoperineal Resection: A Case Report

**DOI:** 10.7759/cureus.113016

**Published:** 2026-07-20

**Authors:** Cláudia Lima, Filipa Monte, Rui Vieira, Teresa Almeida, Hugo Sequeira

**Affiliations:** 1 General Surgery, Unidade Local de Saúde do Alto Minho, Viana do Castelo, PRT; 2 Plastic and Reconstructive Surgery, Unidade Local de Saúde de Gaia/Espinho, Gaia/Espinho, PRT; 3 Plastic and Reconstructive Surgery, Unidade Local de Saúde de Braga, Braga, PRT

**Keywords:** abdominoperineal resection (apr), autologous flap reconstruction, locally advanced rectal cancer, neovagina, perineal reconstruction, singapore flap, total neoadjuvant therapy, vaginal reconstruction, vram flap

## Abstract

Salvage reconstruction of perineal and vaginal defects following initial flap failure is a challenging and rarely reported clinical scenario. While the vertical rectus abdominis myocutaneous (VRAM) flap is a well-established technique for primary perineal reconstruction, its role as a salvage option following failure of initial flap reconstruction is seldom described. This case report presents a 54-year-old woman diagnosed with low rectal adenocarcinoma (cT4b, N+) with invasion of the sphincter complex, posterior vaginal wall, and rectovaginal fistula. After total neoadjuvant therapy (TNT) consisting of chemoradiotherapy with capecitabine followed by nine cycles of consolidation FOLFOX, the patient underwent abdominopelvic resection with initial vaginal wall reconstruction using bilateral Singapore (pudendal thigh) flaps. Postoperative bilateral flap necrosis at 14 days necessitated revision surgery, during which a pedicled VRAM flap, based on the deep inferior epigastric artery, was used for neovagina reconstruction and pelvic dead space obliteration. Pathological analysis confirmed ypT4bN0 (0/12 lymph nodes), modified Ryan tumor regression grade 2, with lymphovascular and perineural invasion. At a 17-month follow-up, there was no evidence of disease recurrence, and the reconstructed vagina and perineum demonstrated satisfactory healing. To our knowledge, no prior reports describe salvage VRAM reconstruction specifically following bilateral Singapore flap failure for vaginal reconstruction after abdominoperineal resection (APR) in an irradiated field. This case illustrates the application of a reconstructive algorithm for perineal and vaginal defects, highlighting the limitations of fasciocutaneous flaps in irradiated fields and the versatility of the VRAM flap as a reliable salvage option when initial reconstruction fails.

## Introduction

Perineal reconstruction following oncologic resection presents distinct challenges due to the anatomical complexity of the region, the frequent use of neoadjuvant radiation, and the resultant non-collapsible pelvic dead space, all of which contribute to high rates of wound healing complications [1,2]. Perineal wound complications after chemoradiotherapy and abdominoperineal resection (APR) for anorectal cancer occur in up to 60% of patients, including perineal abscess, wound dehiscence, and fluid collections [3]. In irradiated fields, primary closure has been associated with significantly higher rates of major perineal wound complications compared with flap-based reconstruction. Devulapalli et al. demonstrated that primary closure was more than twice as likely to be associated with total perineal wound complications compared with flap closure (OR 2.17; 95% CI 1.34-3.14; p = 0.001), with rates of major complications also significantly higher in the primary closure group (OR 3.64; 95% CI 1.43 7.79; p = 0.005) [4].

The vertical rectus abdominis myocutaneous (VRAM) flap is a widely recognized and reliable option for perineal and pelvic reconstruction [5]. Classified as a Mathes and Nahai Type III muscle flap, the VRAM is supplied by dual dominant pedicles from the superior and inferior deep epigastric arteries [6]. When used for perineal reconstruction, the flap is typically based on the inferior pedicle, allowing transposition of well-vascularized, non-irradiated tissue into the pelvis and perineum [7]. Its consistent blood supply, adequate tissue volume, and large skin paddle render it particularly suitable for obliterating pelvic dead space and resurfacing perineal skin defects [8].

While the VRAM flap is well established as a primary reconstructive option, its use as a salvage technique following failure of initial flap reconstruction in the perineum is rarely reported. The Singapore (pudendal thigh) flap is a fasciocutaneous alternative commonly used for vaginal reconstruction, but its reliability in irradiated fields and for extensive defects is limited [9,10]. When initial reconstruction with local fasciocutaneous flaps fails, the reconstructive surgeon must escalate to a more robust option - a concept aligned with the reconstructive ladder or elevator paradigm. This case report describes the successful use of a pedicled VRAM flap as a salvage procedure following bilateral Singapore flap necrosis after APR for locally advanced rectal cancer, highlighting the reconstructive decision-making process and technical considerations in this uncommon clinical scenario. This case adds value beyond standard VRAM reconstruction by documenting the specific decision-making process, technical adaptations, and outcomes when the VRAM is employed as a secondary salvage procedure in a previously operated, irradiated perineal wound - a scenario that presents distinct challenges compared with primary VRAM reconstruction.

## Case presentation

A 54-year-old woman presented with severe anemia (hemoglobin 5 g/dL) and rectal bleeding. A diagnosis of low rectal adenocarcinoma was confirmed through colonoscopy with biopsy. Pelvic magnetic resonance imaging (MRI) revealed a large anorectal tumor invading the sphincter complex, perianal region, posterior vaginal wall, and introitus. A rectovaginal fistula was identified at the eight o'clock position in the lower third of the anal canal. The tumor was staged as cT4b, N+, with positive extramural vascular invasion (EMVI+) and involvement of the mesorectal fascia (MRF+) on MRI. Staging workup showed no evidence of distant metastases.

The patient underwent total neoadjuvant therapy (TNT) in accordance with current guidelines for locally advanced rectal cancer. The treatment protocol consisted of long-course pelvic radiotherapy (50.4 Gy in 28 fractions) with concurrent capecitabine, completed in June 2023, followed by nine cycles of consolidation FOLFOX, completed in November 2023. A restaging pelvic MRI performed in September 2023 (after chemoradiotherapy and during consolidation chemotherapy) demonstrated significant tumor size reduction, although the rectovaginal fistula remained stable.

**Figure 1:**
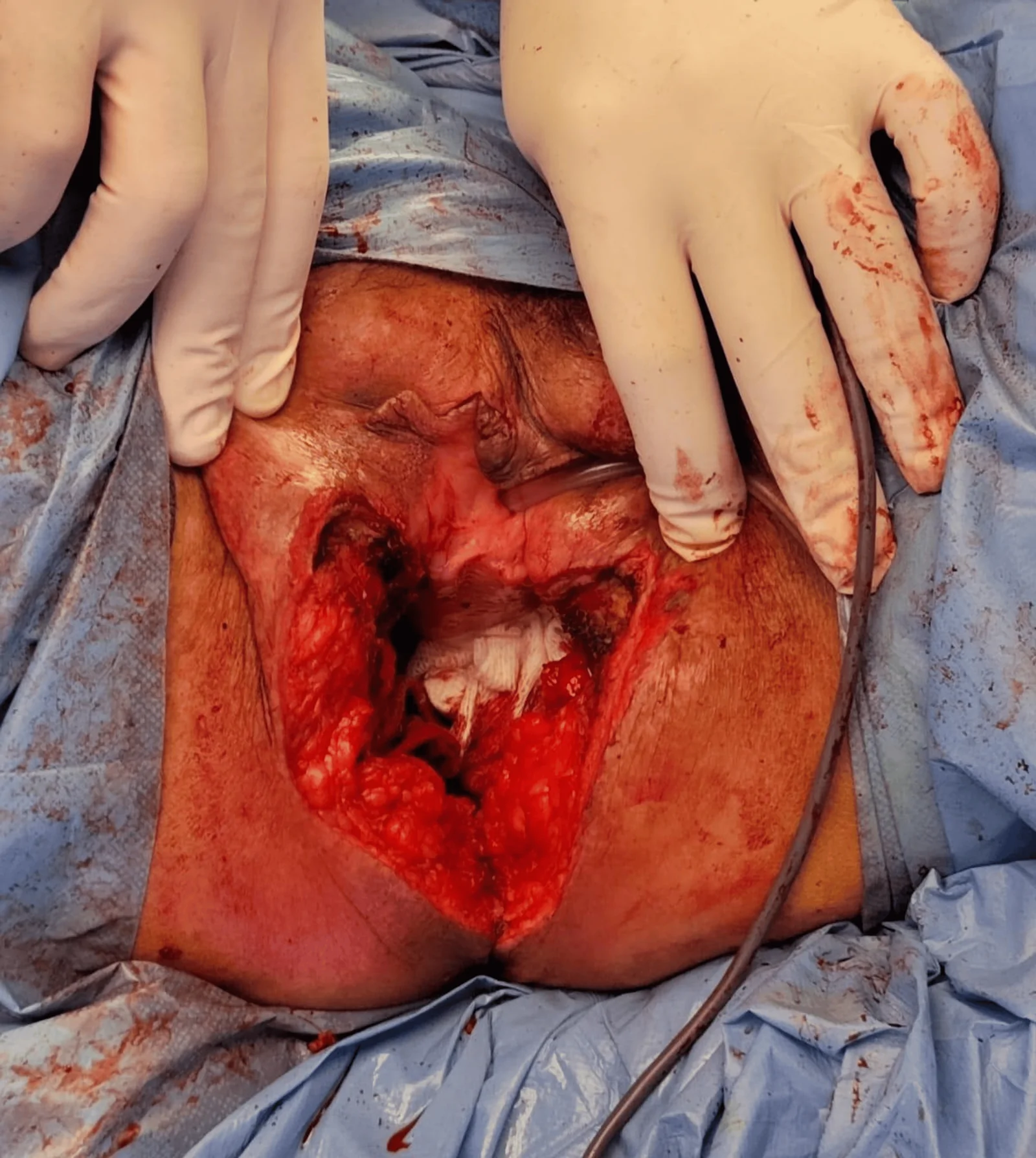
Intraoperative perineal and vaginal defect.

**Figure 2:**
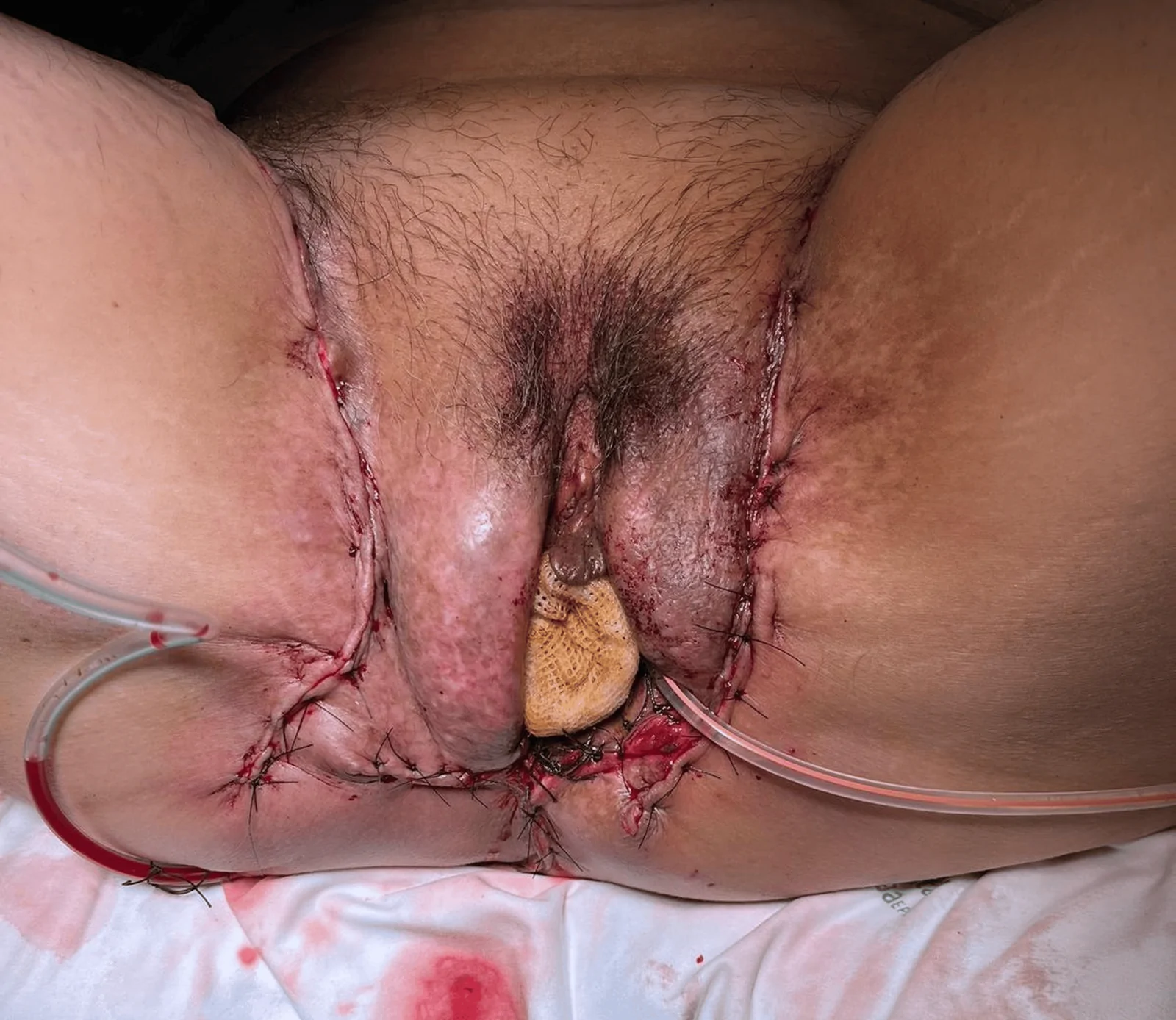
Immediate postoperative Singapore flap reconstruction.

On January 29, 2024, the patient underwent abdominopelvic resection with en bloc removal of the involved posterior vaginal wall. Given the resulting Type IB vaginal defect (partial defect involving the posterior vaginal wall), bilateral Singapore (pudendal thigh) flaps were initially selected for vaginal reconstruction (Figures 1-2). This choice was based on their established reliability for posterior vaginal wall defects, ease of harvest, minimal donor-site morbidity, and the goal of preserving the rectus abdominis muscle and anterior abdominal wall integrity for potential future use, particularly given the presence of a permanent colostomy. Each Singapore flap was designed in the labiocrural fold, measuring approximately 10 × 5 cm, centered over the posterior labial artery pedicle. The flaps were raised in a subfascial plane, including skin, subcutaneous tissue, and deep fascia of the medial thigh. Bilateral flaps were sutured together to reconstruct the posterior vaginal wall, and the donor sites were closed primarily. Postoperative flap monitoring was performed clinically every four hours during the first 48 hours, assessing color, capillary refill, temperature, and turgor.

**Figure 3:**
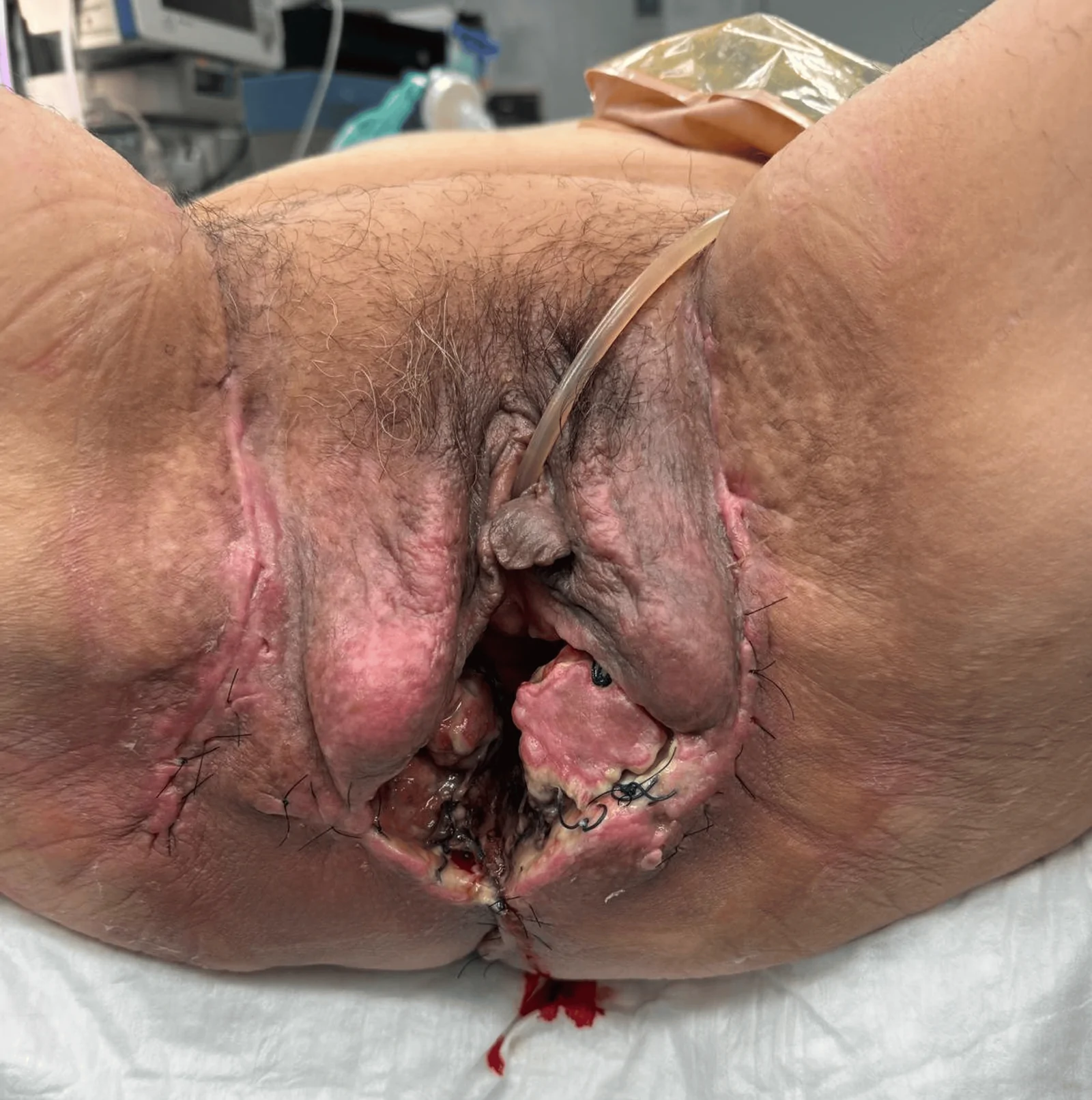
Singapore flap reconstruction showing wound dehiscence.

Fourteen days postoperatively, bilateral flap necrosis and wound dehiscence were detected, with purulent drainage from the perineal wound (Figure 3).

**Figure 4:**
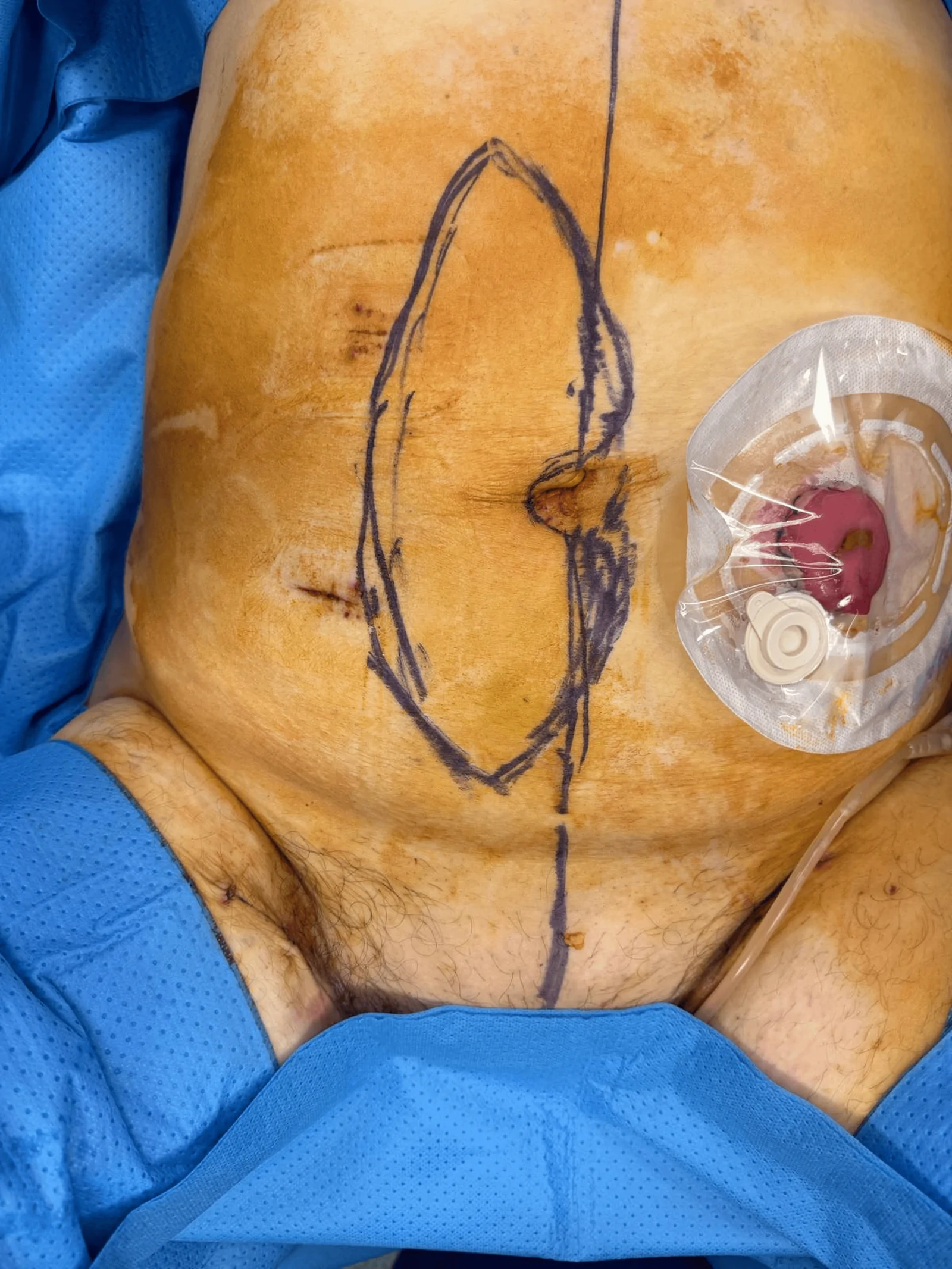
Preoperative markings for the vertical rectus abdominis myocutaneous (VRAM) flap harvest.

Revision surgery was performed on February 12, 2024. During this procedure, all necrotic tissue was debrided from the perineal wound and the vaginal cavity. A right VRAM flap was then harvested from the anterior abdominal wall, contralateral to the left-sided end colostomy, to preserve abdominal wall integrity around the stoma and reduce the risk of parastomal hernia. The skin paddle was designed vertically, measuring approximately 10 × 22 cm, centered over the right rectus abdominis muscle. The flap was raised on the deep inferior epigastric artery pedicle (Figure 4).

**Figure 5:**
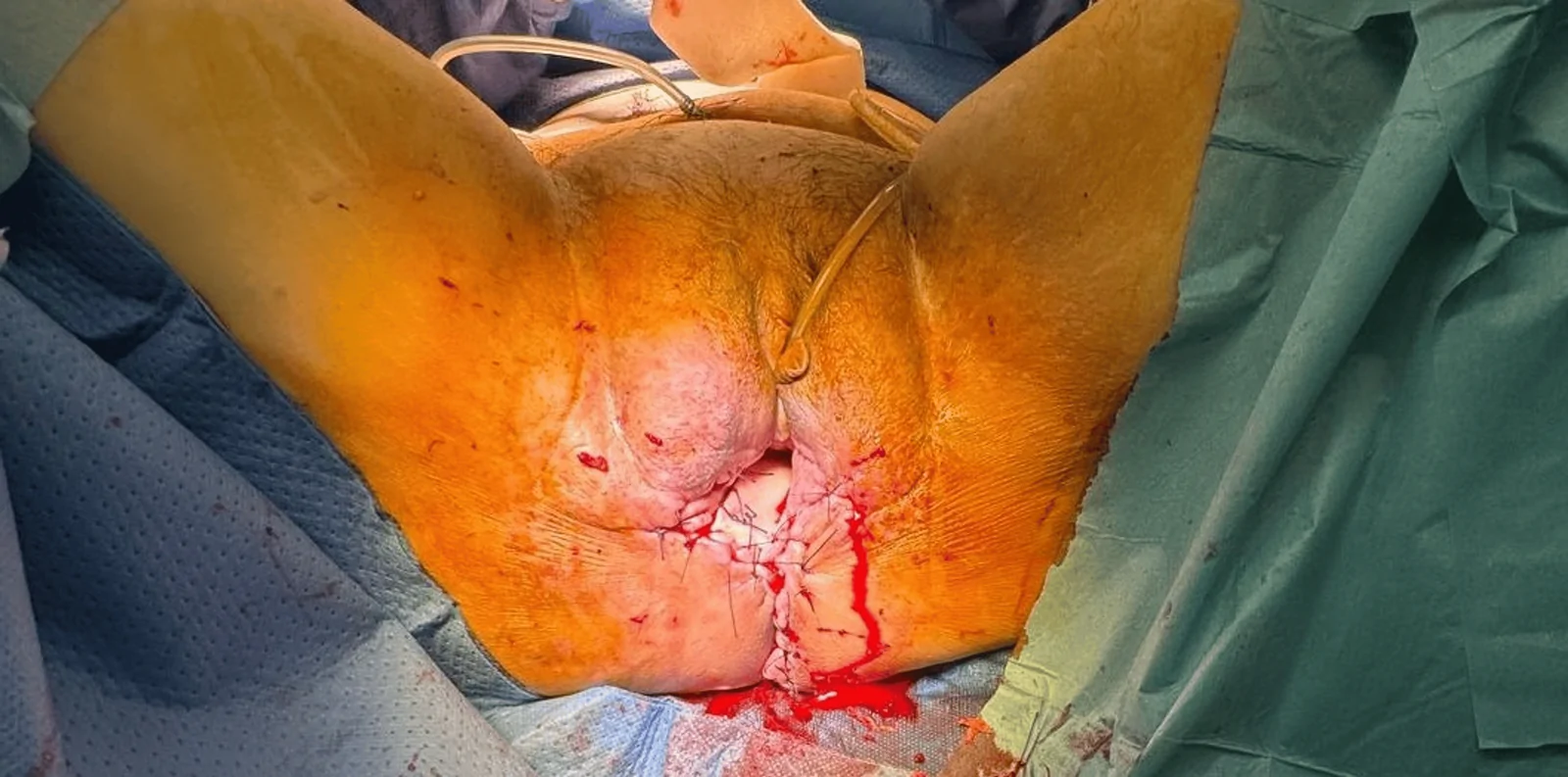
Immediate postoperative VRAM flap reconstruction.

**Figure 6:**
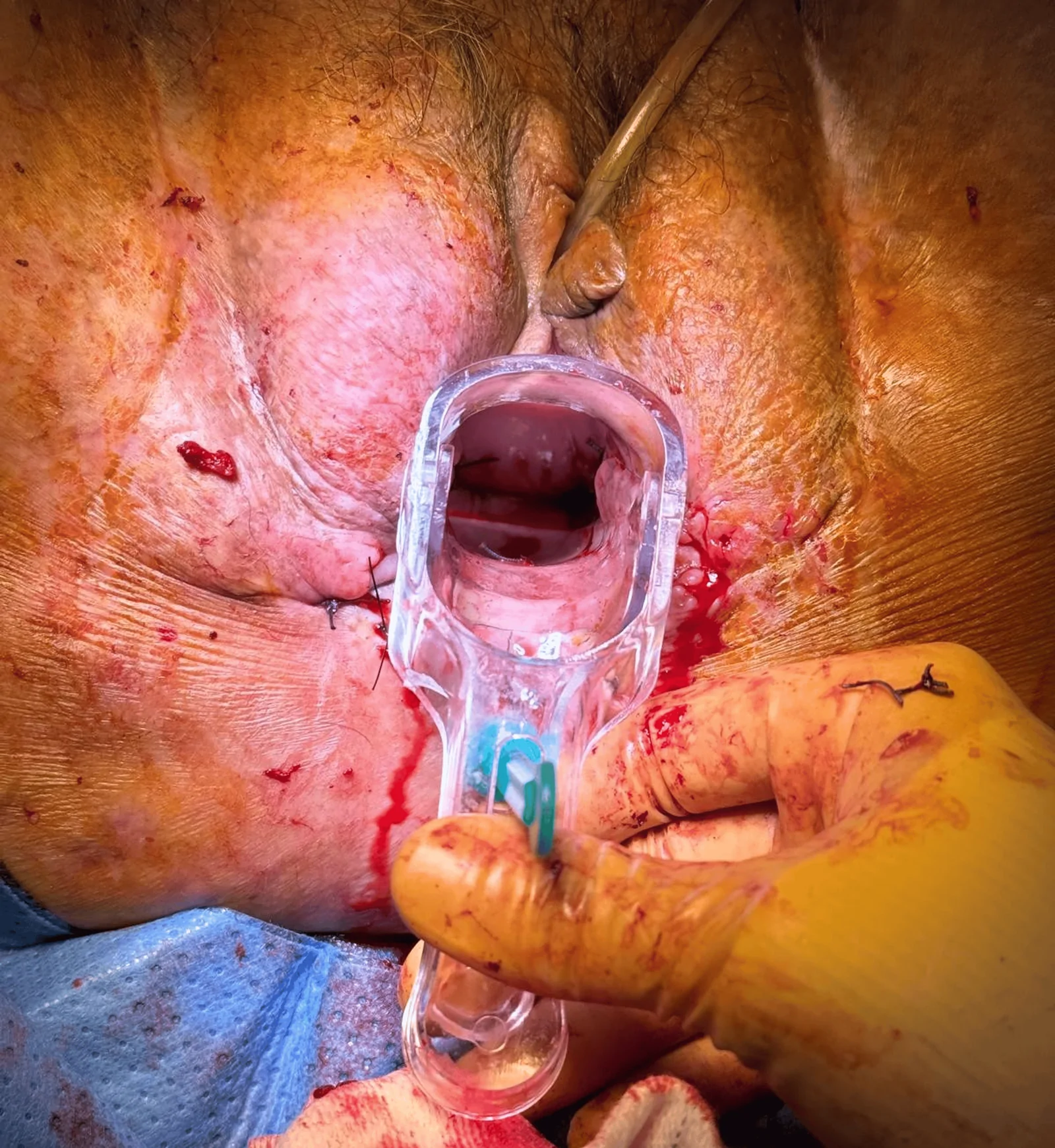
Postoperative vaginal reconstruction.

The anterior rectus sheath was incised, preserving a 1-cm fascial cuff laterally to facilitate closure. The rectus abdominis muscle was divided superiorly, and the superior epigastric vessels were ligated. The flap was tunneled intraperitoneally through the pelvic inlet. For flap transposition into the posterior perineal defect, the VRAM was rotated 180° in the coronal plane, positioning the cranial portion of the flap to cover the most posterior aspect of the defect while avoiding pedicle torsion or tension. The deepithelialized proximal portion of the flap was used to obliterate the pelvic dead space, while the skin paddle, together with viable remnants of the Singapore flaps, was used to reconstruct the posterior vaginal wall and create a neovagina (Figures 5-6).

The donor site was closed in layers. The anterior rectus sheath was reapproximated primarily using a running looped polydioxanone (PDS) suture, with component separation of the external oblique aponeurosis performed on the ipsilateral side to reduce fascial tension. A closed suction drain was placed in the subcutaneous space. No prosthetic mesh was used for donor-site reinforcement. Postoperative VRAM flap monitoring was performed clinically every four hours for the first 72 hours and every eight hours thereafter, assessing skin paddle color, capillary refill, temperature, and turgor. A handheld Doppler was used to confirm pedicle flow at each assessment during the first 48 hours.

Pathological analysis of the resection specimen revealed invasive adenocarcinoma, moderately differentiated (G2), with pathological staging of ypT4bN0. Lymph node status showed zero out of 12 lymph nodes positive for metastatic disease. Tumor regression grade was Modified Ryan Score 2 (minimal response: residual cancer remaining, but with predominant fibrosis). Lymphovascular invasion and perineural invasion were present. The distal margin was 1 cm (negative), and the radial margin was 0.5 cm (negative, as a positive circumferential resection margin is defined as tumor ≤1 mm from the margin).

After 42 days of hospitalization, the patient was discharged in stable condition with a well-healed perineal wound and viable VRAM flap. No donor-site complications (wound dehiscence, hernia, or bulge) were observed during the hospitalization period. At the most recent follow-up (17 months postoperatively), there was no clinical or radiological evidence of disease recurrence, and the reconstructed vagina and perineum demonstrated satisfactory healing. No incisional or parastomal hernia was detected on clinical examination.

## Discussion

This case illustrates the successful application of a pedicled VRAM flap as a salvage reconstructive option following bilateral Singapore flap failure after APR for locally advanced rectal cancer. While the VRAM flap is well established for primary perineal reconstruction, its use as a salvage technique after initial flap failure in this anatomical region is rarely described [5,7,8]. A targeted literature search identified Yonemitsu et al. as the only comparable report of salvage VRAM reconstruction in the perineal region; however, in that case, the indication was recurrent perineal hernia after APR following failed mesh repairs, rather than vaginal reconstruction following fasciocutaneous flap necrosis [11]. No reports describing salvage VRAM specifically following Singapore flap failure for vaginal reconstruction were identified. The present case, therefore, represents, to our knowledge, the first report of this specific clinical scenario.

Vaginal defects resulting from oncologic resection are classified according to the system described by Cordeiro et al. into partial (Type I) and circumferential (Type II) defects [12,13]. The present patient had a Type IB defect (posterior vaginal wall), which, according to this classification, is optimally reconstructed with a rectus abdominis myocutaneous flap [12]. The initial choice of bilateral Singapore flaps was a reasonable alternative given their established role in vaginal reconstruction, ease of dissection, and minimal donor-site morbidity [14]. However, the subsequent bilateral flap necrosis necessitated escalation within the reconstructive algorithm to a VRAM flap, which provided the necessary tissue volume, reliable vascularity from a non-irradiated donor site, and sufficient bulk to obliterate the pelvic dead space. This case highlights an important principle: in irradiated fields with extensive defects, the reconstructive surgeon should have a low threshold for selecting myocutaneous flaps as the primary reconstructive option.

Several factors likely converged to produce bilateral Singapore flap necrosis. The pudendal thigh (Singapore) flap is a fasciocutaneous flap based on the posterior labial artery [15]. Tham et al. demonstrated through cadaveric studies that the flap is a three-vascular-territory flap with the pedicle situated close to the midline; when large flaps are raised, the distal portions have a potentially precarious blood supply, predisposing to apical necrosis [9]. In this patient, three factors converged to produce bilateral flap necrosis: (1) prior pelvic irradiation (50.4 Gy) caused chronic vascular injury, endarteritis obliterans, fibrosis, and impaired angiogenesis in the recipient bed; (2) the extent of the posterior vaginal wall defect required large flaps that exceeded the reliable vascular territory of the Singapore flap; and (3) the distal portions of these large flaps, which depend on the most peripheral branches of the posterior labial artery, were therefore most vulnerable to ischemia - particularly in a recipient bed with radiation-compromised vascularity [16]. This combination of a compromised recipient bed and flaps raised beyond their reliable vascular territory likely explains the bilateral necrosis observed at 14 days. These findings are consistent with the literature: Gleeson et al. reported partial apical necrosis in 4/8 patients and complete bilateral necrosis in 1/8 patients with pudendal thigh flaps, with particularly poor outcomes in irradiated fields [10]. Casey et al. reported a flap-specific complication rate of 37.8% for Singapore flaps compared with 22% for VRAM flaps in a comparative study of 99 vaginal reconstructions [14].

Several technical aspects of the salvage VRAM reconstruction merit discussion. The flap was harvested contralateral to the colostomy to preserve abdominal wall integrity around the stoma and reduce the risk of parastomal hernia. The skin paddle dimensions (approximately 10 × 22 cm) provided adequate tissue for both vaginal reconstruction and pelvic dead space obliteration. For posterior perineal defects, Trapero et al. recommend rotating the VRAM flap 180° in the coronal plane to avoid pedicle torsion, positioning the cranial part of the flap to cover the most posterior aspect of the defect [17]. This technique was employed in the present case with successful flap transposition. Donor-site closure was achieved with primary fascial reapproximation augmented by ipsilateral component separation, without prosthetic mesh. Baumann and Butler demonstrated that component separation resulted in fewer postoperative wound complications, hernias (6% vs. 24%), and bulges compared with primary fascial closure alone when excessive tension was present [18]. Vervaet et al., in a systematic review of 2,200 patients, reported incisional hernia in 10.2% and parastomal hernia in 19.2% following VRAM harvest over a mean follow-up of 31 months [19]. In the present case, no donor-site hernia was detected at 17 months, although longer follow-up is warranted.

This is a single case report, and the findings cannot be generalized. The follow-up period of 17 months, while encouraging, is insufficient to draw definitive conclusions regarding long-term oncologic outcomes, donor-site hernia rates, or functional vaginal outcomes. Formal assessment of sexual function and quality of life was not performed, and no validated patient-reported outcome measures (PROMs) were used to objectively assess functional outcomes, body image, or quality of life, which limits the assessment of the reconstruction's impact beyond clinical and anatomical endpoints.

## Conclusions

This case illustrates the successful use of a pedicled VRAM flap as a salvage reconstructive option following bilateral Singapore flap failure after APR for locally advanced rectal cancer in an irradiated field. The case underscores several important points: (1) the importance of a reconstructive algorithm for vaginal and perineal defects, with the VRAM flap serving as a reliable salvage option; (2) the limitations of Singapore flaps for extensive posterior vaginal defects in irradiated fields, where their precarious distal blood supply and limited tissue bulk may be insufficient; and (3) the technical considerations for salvage VRAM transposition, including contralateral harvest, coronal plane rotation, and donor-site closure with component separation. Longer follow-up is needed to assess durable oncologic and functional outcomes.
